# Saturation of long-term potentiation in the dorsal cochlear nucleus and its pharmacological reversal in an experimental model of tinnitus

**DOI:** 10.1016/j.expneurol.2017.02.011

**Published:** 2017-06

**Authors:** Thomas Tagoe, Daniel Deeping, Martine Hamann

**Affiliations:** Department of Neurosciences, Psychology and Behaviour, University of Leicester, UK

**Keywords:** DCN, dorsal cochlear nucleus, EPSC, excitatory post-synaptic potential, HFS, high frequency stimulations, LTP, long-term potentiation, Mg^2 +^,  magnesium, NMDA, *N*-methyl-d-aspartate, PPR, paired pulse ratio, PSFP, post-synaptic field potential, Synaptic plasticity, Long-term potentiation, Auditory, Synapse, Central auditory system, Release probability

## Abstract

Animal models have demonstrated that tinnitus is a pathology of dysfunctional excitability in the central auditory system, in particular in the dorsal cochlear nucleus (DCN) of the brainstem. We used a murine model and studied whether acoustic over-exposure leading to hearing loss and tinnitus, affects long-term potentiation (LTP) at DCN multisensory synapses. Whole cell and field potential recordings were used to study the effects on release probability and synaptic plasticity, respectively in brainstem slices. Shifts in hearing threshold were quantified by auditory brainstem recordings, and gap-induced prepulse inhibition of the acoustic startle reflex was used as an index for tinnitus. An increased release probability that saturated LTP and thereby induced metaplasticity at DCN multisensory synapses, was observed 4–5 days following acoustic over-exposure. Perfusion of an NMDA receptor antagonist or decreasing extracellular calcium concentration, decreased the release probability and restored LTP following acoustic over-exposure. In vivo administration of magnesium-threonate following acoustic over-exposure restored LTP at DCN multisensory synapses, and reduced gap detection deficits observed four months following acoustic over-exposure. These observations suggest that consequences of noise-induced metaplasticity could underlie the gap detection deficits that follow acoustic over-exposure, and that early therapeutic intervention could target metaplasticity and alleviate tinnitus.

## Highlights

• Acoustic over exposure increases the release probability and saturates long-term potentiation in the rat dorsal cochlear nucleus
• Acoustic over exposure triggers gap detection deficits, an experimental model of tinnitus
• Administration of magnesium-threonate following acoustic over-exposure restores long-term potentiation and reduces gap detection deficits

## 1 Introduction

innitus, the pathological percept of phantom sound, affects 10 to 15% of the adult population worldwide (Dawes et al., 2014, Shargorodsky et al., 2010). Tinnitus has been shown to correlate with aberrant neural activity in the dorsal cochlear nucleus (DCN) (Kaltenbach, 2007), the first relay in the auditory brainstem integrating acoustic and multimodal sensory inputs. Tinnitus is still a poorly understood auditory percept with studies suggesting that altered excitability in the DCN initiates a complex sequence of events relayed to higher levels of the auditory pathway (Brozoski et al., 2002, Ma et al., 2006). For example, acoustic overexposure triggering hearing loss and tinnitus has been shown to enhance DCN somatosensory and vestibular synaptic inputs (Barker et al., 2012, Shore et al., 2008) supporting the idea that tinnitus arises in response to enhanced multisensory synaptic transmission to the DCN (Shore et al., 2008).

Tinnitus has been defined as a pathology of synaptic plasticity in the central auditory pathway (Guitton, 2012, Tzounopoulos, 2008). Synaptic plasticity describes alteration in synaptic strength among connected neurons: this can be either increased, as observed with long-term potentiation (LTP); or decreased, as in long-term depression (LTD) (Bear and Malenka, 1994, Bliss and Collingridge, 1993, Malenka and Bear, 2004). Synaptic plasticity itself is subject to activity-dependent variation as it can be dynamically regulated by prior activity, in a process termed ‘metaplasticity’ (Abraham, 2008). Aberrant plasticity or metaplasticity has been implicated in the pathophysiology of autism spectrum disorder and fragile X syndrome (Oberman et al., 2016). Recent studies also demonstrated links between chronic pain and metaplasticity promoting excessive amplification of ascending nociceptive transmission to the brain (Li and Baccei, 2016), and between persistent LTP inhibition and memory impairment in Alzheimer's disease (Jang and Chung, 2016).

Whereas the presence of LTP has been demonstrated in the DCN (Tzounopoulos et al., 2004), direct evidence demonstrating metaplasticity in response to acoustic over-exposure triggering tinnitus has yet to be provided. Here we investigate the effect of acoustic over-exposure on plasticity at DCN multisensory synapses and a potential therapeutic reversal of this effect that also ameliorates perception of tinnitus.

## 2 Materials and methods

One hundred and eight Wistar rats (male and female) were used. Experiments were performed in accordance with the UK Animals (Scientific Procedures) Act of 1986 Home Office regulations and approved by the Home Office and Leicester University Ethical Committee (PIL 80/8158, PPL 60/4351).

### 2.1 Acoustic over-exposure

Rats were aged P15-P18 at the first day of acoustic over-exposure, which corresponds to the period after hearing onset (Geal-Dor et al., 1993). Rats were anesthetised with an intraperitoneal injection of fentanyl (0.15 mg/kg), fluanisone (5 mg/kg, VetaPharma Ltd) and Hypnovel (2.5 mg/kg, Roche). Using this combination of anaesthetics, rats were initially anesthetised for about 1 h, after which animals stayed sedated. Rats were placed in a custom made open field sound-insulated chamber containing a 600 W High Power Horn Tweeter radiating evenly, frequency range 2–20 kHz (Maplin UK) so that both ears were exposed. Bilateral noise exposure was used as it best approximates the noise exposure that occurs in humans (Metidieri et al., 2013). A pure tone of 14.8 kHz was delivered at 110 dB SPL for a total of 9 h (3 h per day over 3 consecutive days) as previously described (Tagoe et al., 2014). Age-matched control animals from the same litter were similarly anesthetized but unexposed to acoustic over-exposure. In vitro auditory brainstem recordings or gap detection screening following the acoustic over-exposure or the anaesthesia only were performed blind.

### 2.2 Auditory brainstem response recordings

Rats were anesthetised using similar anaesthetics as mentioned above. Auditory brainstem response recordings were performed at three time points: before, 4 days, and 18 weeks after anaesthesia only (controls) or after acoustic over-exposure. Positive, negative, and ground electrodes were inserted subcutaneously at the vertex, mastoid, and back, respectively (Pilati et al., 2012b). Auditory brainstem responses were evoked by calibrated tone pips (8, 16, 24, 30 kHz; 1 ms rise and fall times, 5 ms duration, 3 ms plateau) generated in a free field at 10 Hz by a waveform generator (TGA 1230 30 MHz, Tucker Davis Technology, USA) and an acoustic driver (Bruel & Kjaer type 4192, Denmark). Evoked responses were recorded by an amplifier (Medelec Sapphire 2A, Oxford Instruments, UK), band-pass filtered between 10 Hz and 5 kHz and averaged from 300 to 400 Hz sweeps or 800 to 1000 sweeps at threshold using a custom made software (CAP, GSK). Tone pips were progressively attenuated in 10 or 3 dB SPL steps from an initial intensity of 94 dB SPL using a digital attenuator (PA4, Tucker Davis Technology, USA). Hearing thresholds were defined as the lowest sound pressure level at which peaks 1 and 2 could be recognized (Barker et al., 2012, Pilati et al., 2012a, Tagoe et al., 2014). Detection of peaks was confirmed by comparing the auditory brainstem waveform with two or three suprathreshold waveforms. Final determination of threshold was made by reanalysing the traces off-line. Threshold shifts were used as the primary indicator of hearing performance and were measured at the left ear as the difference between the hearing threshold on day 1 (P15–18) and the hearing threshold 4 days after the acoustic over-exposure procedure.

### 2.3 Behavioural assessment of tinnitus

The behavioural assessment of tinnitus is based on the gap detection paradigm originally described by (Turner et al., 2006). The paradigm is based on the pre-pulse inhibition of the acoustic startle reflex whereby the startle reflex is inhibited by a short silent gap embedded in a continuous background noise. Turner et al. (2006) demonstrated selective gap detection deficits in rats following acoustic over-exposure that they hypothesised were due to tinnitus. Gap detection deficits were assessed using a specific acoustic startle reflex hardware and software (Kinder Scientific, Poway, CA). Each rat was presented with a constant 65 dB SPL background noise consisting of octave based sounds centred at either 8 kHz, 16 kHz, 24 kHz, 30 kHz or broadband noise (BBN). A 110 dB SPL, 20 ms BBN noise burst served as the startle stimulus to induce the acoustic startle reflex. During the background noise, the rat was either presented with the startle stimulus alone (startle only condition) or the startle stimulus preceded by a silent gap embedded within the background noise (GAP condition). Silent gaps (50 ms in duration with a 0.1-ms rise/fall) began 100 ms before the startle stimulus. Each testing session began with a 2-minute acclimatisation period to the background sound. This was followed by two trials of startle stimuli to trigger initial startle reflexes that were excluded from the analysis. The testing phase consisted of mixing a pseudo-random sequence of 12 startle only trials (with no silent gaps) with 12 trials containing a silent gap, both embedded in similar background noise preceding the startle stimulus. Startle responses were converted into gap detection ratios (GDRs) whereby for a given frequency, the mean startle response to the gap condition was divided by the mean startle only response. Screening was first performed at P15-P18 where startle response amplitudes were compared in the presence and absence of gaps embedded in broadband noise. This allowed selecting rats displaying an ability to detect gaps prior to the original testing phase. Selected rats were then randomly assigned to either a control or an exposed group and screening was repeated 18 weeks following acoustic over-exposure or anaesthesia only. Auditory brainstem response recordings were used to confirm recovery from hearing loss 18 weeks following acoustic over-exposure, ensuring that the effects on gap detection deficits were specific rather than due to hearing loss.

### 2.4 Magnesium administration

Magnesium was administered by supplementing normal drinking water with Mg^2 +^-threonate (604 mg/kg/day corresponding to 50 mg/kg/day elemental Mg^2 +^ (Abumaria et al., 2011)) on the last day of the acoustic over-exposure for a maximal period of 18 weeks. The dose has previously been shown to be effective in elevating brain Mg^2 +^ (Slutsky et al., 2010).

### 2.5 Multisensory input stimulation

Multisensory inputs to the DCN were stimulated by placing a bipolar stimulating electrode (FHC Inc., USA) in the molecular layer (Oertel and Young, 2004). Field potential and whole cell recordings were performed in the dorsal segment of the fusiform cell layer encoding high frequencies (Muniak and Ryugo, 2014) as previously described (Tagoe et al., 2014).

### 2.6 Field potential recordings

**Fig. 1:**
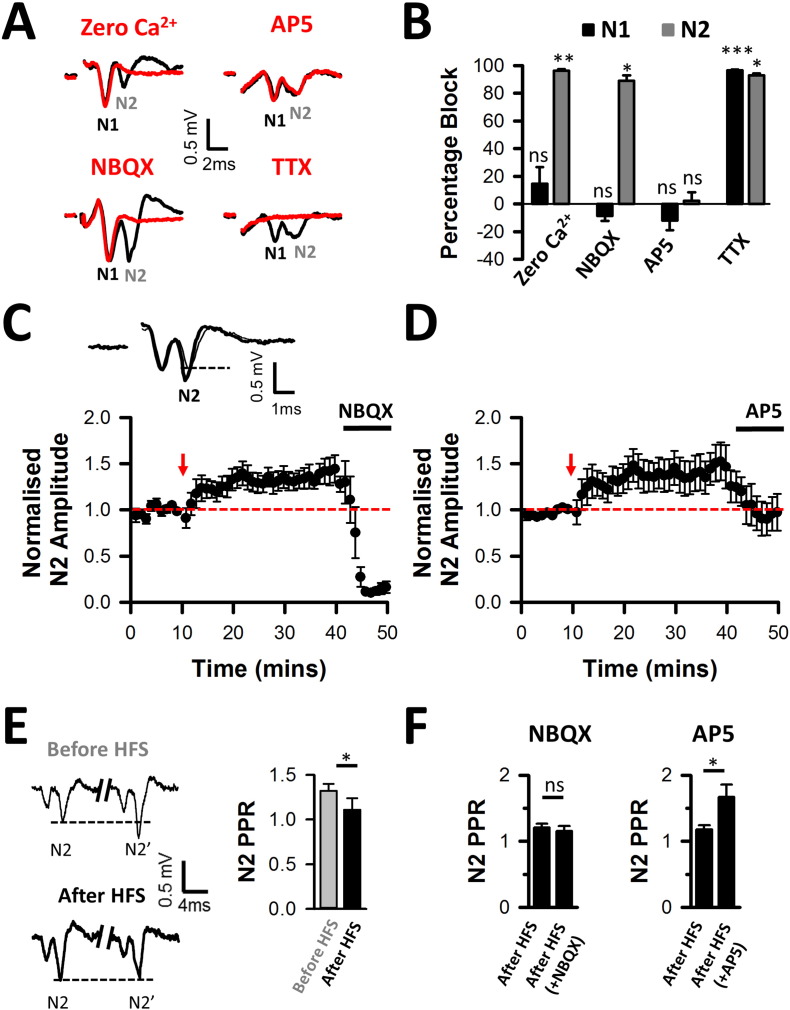
Induction of presynaptic LTP and increased paired pulse facilitation at dorsal cochlear nucleus multisensory synapses. Field potentials were recorded in normal extracellular medium (black) and in test conditions (red) as indicated. Changing the perfusion medium to a zero Ca^2 +^ solution or perfusing NBQX (10 μM) blocked the postsynaptic field potential (PSFP, N2) without affecting the presynaptic component of the field potential (N1). Perfusing TTX (1 μM) abolished N1 and consequently the PSFP (N2). Stimulation artefacts were deleted for clarity. (B) Bar charts summarizing the effects shown in ‘A’ on N1 (black) and on the PSFP (N2, grey). N1 was abolished by TTX (n = 6, N = 5); N2 was abolished by TTX (n = 6, N = 5), 0 mM Ca^2 +^ (n = 7, N = 7) and NBQX (n = 7, N = 6). D-AP5 (25 μM) failed to affect either N1 or N2 (n = 10, N = 8). *P < 0.05, **P < 0.01, ***P < 0.001, paired *t*-tests. (C) High frequency stimulations (red arrow) induce LTP, increasing the normalised PSFP amplitudes from 1.00 ± 0.16 to 1.39 ± 0.09 (n = 7, N = 5; Z = 3.46, P < 0.001, Wilcoxon). Perfusion of NBQX (10 μM) abolished the PSFP (and the LTP). Inset: Sample average traces recorded before high frequency stimulations (thin line) and during LTP (thick line). The dotted horizontal line represents the peak amplitude before high frequency stimulations. (D) Induction of LTP by high frequency stimulations and its inhibition by D-AP5 (25 μM) returning PSFP amplitudes to baseline levels (PSFP normalised amplitudes decreasing from 1.58 ± 0.17 to 1.02 ± 0.18 (n = 8, N = 6, Z = − 2.02, P = 0.04, Wilcoxon). (C, D) The red dashed line indicates the baseline amplitude and the red arrow points to the time of high frequency stimulations. (E left) Example average traces showing paired pulse facilitation before high frequency stimulations (upper traces) and an absence of paired pulse facilitation during LTP (lower traces). N2 and N2’ represent the first and the second PSFP respectively separated by a 60 ms pulse interval. The dashed line points to the amplitude of the first PSFP (N2). (E right) Summary histograms representing paired pulse ratios (N2’ amplitude divided by N2 amplitude) calculated at 60 ms stimulus interval, and showing the decreased paired pulse ratios during LTP (from 1.32 ± 0.08 before high frequency stimulations to 1.11 ± 0.13 during LTP (n = 7, N = 5; Z = − 2.7, P = 0.004, Wilcoxon). (F) Perfusion 0.1 μM NBQX reduces PSFP by 26 ± 8% (n = 4, N = 3, x_r_^2^(2) = 8, P = 0.02, repeated measures (RM) ANOVA on Ranks, SNK test). Histograms showing the absence of effect of 0.1 μM NBQX on paired pulse ratios (F left, n = 4, N = 3, Z = − 0.7, Wilcoxon, ns: non-significant), and the increased paired pulse ratios (paired pulse facilitation) in the presence of 25 μM AP5 (from 1.17 ± 0.07 to 1.67 ± 0.19, n = 8, N = 6; Z = 2.24, P = 0.023, F right).

Our study took advantage of field potential recordings to allow stable and prolonged recordings from a large number of undialysed cells in the DCN fusiform cell layer, including fusiform, granule and cartwheel cells (Oertel and Young, 2004). Using field potentials also limited the risk of washing out intracellular substances that could be essential for studying LTP and metaplasticity (Abrahamsson et al., 2016). This proved beneficial, as we were able to record LTP for at least 60 min and perform various experimental procedures within that time-window. Coronal brainstem slices (250 μm) containing the DCN were obtained from rats 4–5 days after acoustic over-exposure (or anaesthesia only) between P19 and P23, and also 1 month after acoustic over-exposure between P49 and P54. Dissection of the brainstem and slicing procedures were performed as previously described (Barker et al., 2012, Pilati et al., 2012a). Field potential recordings were performed in normal extracellular solution containing (in mM): NaCl 125, KCl 2.5, NaH_2_PO4 1.2, d-glucose 10, ascorbic acid 0.5, Na pyruvate 2, myo-inositol 3, NaHCO_3_ 26, CaCl_2_ 2 and MgCl_2_ 0.1. Parallel fiber evoked field potentials recorded in the DCN fusiform layer is a composite of events with nomenclature which has been described previously (Manis, 1989). The amplitude of the N1 or the PSFP (N2) wave was measured as the negative peak amplitude minus the baseline amplitude measured by interpolation. The contributions of pre- and postsynaptic components of the field potentials were determined as described in Fig. 1A and B. Paired pulse facilitation was assessed at a paired pulse interval of 60 ms. LTP was induced by applying a high frequency stimulation (HFS: 50 Hz for 30s) (Grover and Teyler, 1994) and represented as increased PSFP amplitudes normalised to the average PSFP amplitude over the last 5 min prior to HFS.

### 2.7 Whole cell patch clamp recordings

Coronal brainstem slices (180 μm) containing the DCN were obtained from Wistar rats (Barker et al., 2012, Pilati et al., 2012a). Whole cell recordings of fusiform cells were here conducted at 4–5 days after acoustic over-exposure or anaesthesia (i.e. P19–23) as reliable recordings could only be obtained from juvenile rats. Fusiform cells were identified on the basis of morphological and electrophysiological properties as previously described (Pilati et al., 2012b). Whole-cell recordings were made with 3–5 MΩ pipettes filled with Cs-chloride based solution containing (in mM): 120 CsCl, 4 NaCl, 4 MgCl_2_, 0.001 CaCl_2_, 10 Hepes, 2 Mg-ATP, 0.2 GTP (Tris salt), 10 EGTA and 2 QX-314 (All from Sigma). Whole cell recordings were performed using a Multiclamp 700 A amplifier (Molecular Devices Inc. USA), low-pass filtered at 4 kHz and digitized at 20 kHz through a Digidata 1200 interface (Axon Instruments, Foster City, CA), using PClamp 9 software (Molecular Devices Inc. USA). Fusiform cells were held at − 70 mV. Series resistances of < 12 MΩ were compensated by 70%. Excitatory postsynaptic currents (EPSCs) were elicited similarly as above and slices were superfused at 1 ml/min with oxygenated extracellular medium at ~ 33 °C. To allow the isolation of AMPA receptor mediated EPSCs, all recordings were performed in the presence of 20 μM strychnine and 10 μM gabazine to block inhibitory synaptic transmission and 25 μM D-(−)-2-amino-5-phosphonopentanoic acid (D-AP5) to block NMDA receptors. The variance-mean method of quantal analysis was performed by evoking AMPA receptor mediated EPSCs at a sub-maximal stimulation intensity of 0.4 mA and changing Ca^2 +^ concentrations (0.5 mM, 1 mM, 1.25 mM, 1.5 mM, 2 mM, 2.5 mM and 3 mM), as previously described (Tagoe et al., 2014).

### 2.8 Input-output relationships

Input-output relationships were performed for presynaptic and postsynaptic field potential amplitudes, and for fusiform cell EPSC amplitudes, and were fitted with a Hill function as described in (Tagoe et al., 2014).

### 2.9 Statistical analysis

Data distributions were tested for normality using D'Agostino and Pearson omnibus normality tests. Paired or unpaired student *t*-tests were used when distributions were normal. Alternatively, when distributions were not normal or when data had been normalised, the Wilcoxon test was used to test for in-group differences whereas the Mann-Whitney test was used to test for differences between groups. A one way ANOVA test or an ANOVA on Ranks test was used when comparing multiple data sets that were normally or not normally distributed respectively. Those tests were run with Dunn's post hoc tests. Repeated Measures ANOVA on Ranks' test was also used with Student-Newman-Keuls (SNK) post hoc test to assess for differences between more than two data sets at multiple time points. The linear mixed model was also used to identify significant interactions between time and treatment group for gap detection ratios obtained at 8 kHz, 16 kHz and broadband noise (P < 0.05). The linear mixed model was used with a restricted maximum likelihood procedure and a fixed effect test. Prior to using the linear mixed model, the Z-score test was used to identify and remove a single outlier from the data set. Statistics were performed using GraphPad Prism version 5 except for the linear mixed model, which was performed using SPSS version 20. Data are presented as mean ± SEM and considered statistically significant when P < 0.05. N and n represent the number of animals and the number of samples (cells) respectively.

## 3 Results

### 3.1 Presynaptic modulation of LTP at dorsal cochlear nucleus multisensory synapses

We studied the modulation of long-term potentiation in brainstem slices containing the DCN by stimulating parallel fibers in the molecular layer and recording field potentials in the fusiform cell layer, as previously described in (Manis, 1989). Extracellular field potentials produced by DCN parallel fibers and postsynaptic cells (Manis, 1989) comprise two negative waves (Fig. 1A). An initial presynaptic volley (N1) was abolished by a sodium channel blocker, tetrodotoxin (1 μM) (Fig. 1A, & B). A postsynaptic field potential (PSFP: N2) was abolished by an AMPA receptor inhibitor, NBQX (10 μM) or by zero Ca^2 +^ (Manis, 1989), but was unaffected by blocking NMDA receptors with 25 μM D-AP5 (Fig. 1A & B), as predicted for Ca^2 +^-dependent glutamate release activating AMPA receptors under basal stimulation conditions. High frequency stimulations (HFS: 50 Hz for 30 s) induced LTP of the PSFPs (Fig. 1C). As expected from Fig. 1A, blocking AMPA receptors after HFS abolished PSFPs (and therefore LTP) (Fig. 1C). By contrast, blocking NMDA receptors after HFS only abolished the increased PSFP amplitude due to LTP (Fig. 1D).

Most studies on LTP identify presynaptic and postsynaptic mechanisms mediated by AMPA and/or NMDA receptor activation (Fujino and Oertel, 2003, Padamsey and Emptage, 2014, Park et al., 2014). Paired pulse facilitation measurements have been used to distinguish pre- and postsynaptic mechanisms of LTP (Oleskevich et al., 2000). Here we detected decreased paired pulse facilitation (Fig. 1E), providing the first indication that LTP at DCN multisensory synapses was due to an increased release probability. We next tested whether release probability during LTP was affected by blocking AMPA or NMDA receptors. When applied at a sub-maximal dose during LTP, NBQX (0.1 μM) reduced PSFPs but failed to affect paired pulse ratios (Fig. 1F left) as predicted for a postsynaptic inhibition of AMPA receptors (Zucker and Regehr, 2002). By contrast, blocking NMDA receptors during LTP led to an increased paired pulse facilitation (Fig. 1F right), suggesting a decreased release probability, abolishing LTP and returning PSFP amplitudes to baseline levels (Fig. 1D).

**Fig. 2:**
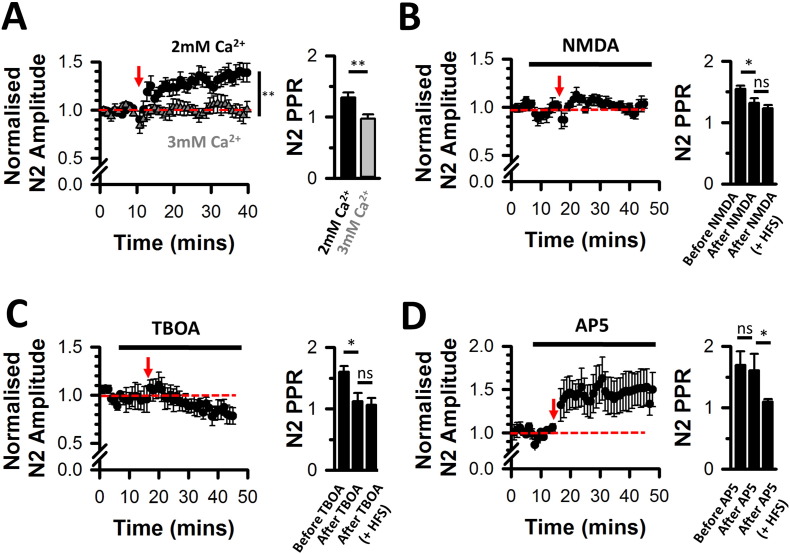
Absence of LTP (metaplasticity) is linked to an absence of paired pulse facilitation prior to the induction protocol. (A left) Presence and absence of LTP in presence of 2 mM and 3 mM extracellular Ca^2 +^ respectively (Mann Whitney test, P < 0.05). (A right) Absence of paired pulse facilitation in presence of 3 mM Ca^2 +^ (n = 5, N = 4 in 3 mM Ca^2 +^, n = 10, N = 4 in 2 mM Ca^2 +^; U = 10; **P = 0.002, Mann Whitney). (B left) Absence of LTP in presence of 500 nM NMDA (n = 5, N = 3). (B right) Decreased paired pulse ratios in presence of NMDA at baseline stimulation rates (n = 5, N = 3; x_r_^2^ (2) = 8.4, P = 0.01, RM ANOVA on Ranks). Subsequent high frequency stimulations failed to affect the paired pulse ratios. (C left) Absence of LTP in presence of 10 μM TBOA (n = 5, N = 3; x_r_^2^ (2) = 2.8, NS, RM ANOVA on Ranks). (C right) Absence of paired pulse facilitation assessed at baseline stimulation rates in presence of 10 μM TBOA (n = 5, N = 3; x_r_^2^ (2) = 7.6, P = 0.01, RM ANOVA on Ranks). Similarly to ‘B’, subsequent high frequency stimulations failed to affect the paired pulse ratios. (D left) Induction of LTP in the presence of 25 μM D-AP5 (normalised PSFP amplitude increase from 1.02 ± 0.02 to 1.52 ± 0.2 (n = 8, N = 5; x_r_^2^ (2) = 7.8, P = 0.03, RM ANOVA on Ranks, SNK test). (D right) Paired pulse facilitation in the presence of D-AP5 at baseline stimulations rates and absence of paired pulse facilitation after LTP was elicited in the presence of D-AP5 (n = 6, N = 5; x_r_^2^ (2) = 6.87, P = 0.029, RM ANOVA on Ranks, SNK test). (B–D): RM ANOVA on Ranks, SNK test, *P < 0.05. ns: non-significant.

We next varied extracellular Ca^2 +^ concentration to directly influence release probability (Oleskevich et al., 2000, Schulz, 1997). Paired pulse facilitation was observed in the presence of 2 mM extracellular Ca^2 +^ concentration but was abolished at 3 mM Ca^2 +^ (Fig. 2A right), confirming the relation between increased release probability and the absence of paired pulse facilitation in our model. Increasing extracellular Ca^2 +^ concentration to 3 mM also abolished LTP induction by HFS (Fig. 2A left), indicating that a low release probability is a prerequisite for LTP induction.

In accordance with our observation that blocking NMDA receptors leads to an increased paired pulse facilitation indicating a decreased released probability (Fig. 1F right), perfusing 500 nM NMDA decreased paired pulse facilitation (Fig. 2B right), suggesting an increased released probability following NMDA receptor activation. The presence of NMDA also prevented the induction of LTP (Fig. 2B left). Therefore, we hypothesised that NMDA receptors act as biosensors for extracellular glutamate, and that their activation leads to an increased release probability to prevent LTP induction. We tested this hypothesis using DL-threo-beta-benzyloxyaspartate (TBOA; a non-transportable antagonist of glutamate uptake) to increase extracellular glutamate concentration at the synapse and delay its clearance (Shimamoto et al., 1998). Similar to the effect of NMDA, TBOA (10 μM) abolished paired pulse facilitation and prevented the induction of LTP (Fig. 2C), Thus LTP induction at DCN multisensory synapses occurs via an NMDA receptor-dependent pathway modulating presynaptic release at these synapses (Fujino and Oertel, 2003, Tzounopoulos et al., 2007).

Previous studies in the DCN have reported that LTP also occurs via an NMDA receptor-independent pathway (Fujino and Oertel, 2003, Tzounopoulos et al., 2007). We also identified an NMDA receptor-independent pathway at DCN multisensory synapses as LTP can be induced in the continuous presence of D-AP5 (Fig. 2D left). A decreased paired pulse ratio occurred alongside LTP in this condition (Fig. 2D right) suggesting that an increased release probability was still underlying LTP expression regardless of the induction pathway. In summary, low release probability is a prerequisite for the induction of LTP at DCN multisensory synapses.

### 3.2 Acoustic over-exposure increases presynaptic release probability and alters the induction of LTP in the DCN

**Fig. 3:**
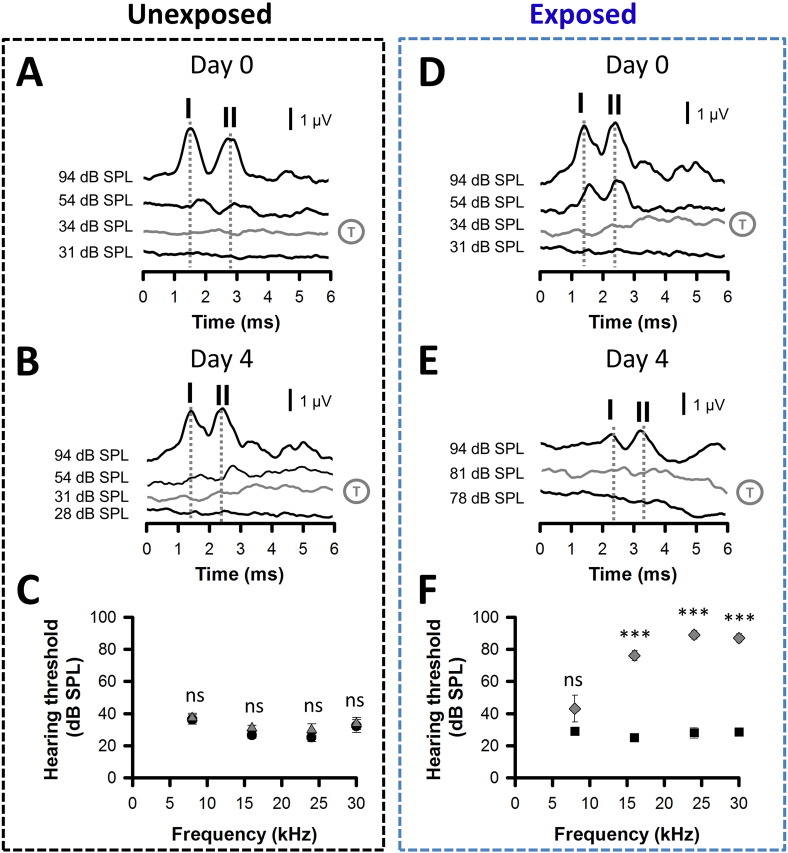
Increased hearing thresholds following acoustic overexposure. (A–C) Hearing thresholds in unexposed conditions. Sample auditory brainstem response (ABR) recordings were obtained from an unexposed rat on day 0 (A) and day 4 (B) of tests. ABR traces were elicited by tone pips at 16 kHz and varying intensities (in dB SPL) as indicated. Traces are shown truncated to 6 ms and waves I and II indicated above the traces elicited at 94 dB SPL. Threshold values (shown as a circled T) were 34 dB SPL on day 0 and 31 dB SPL on day 4 in unexposed conditions. (C) Graph summarizing hearing thresholds recorded at multiple frequencies on day 0 (black circles) and day 4 (grey triangles) (*N* = 6). (D–F) Increased hearing thresholds after acoustic over-exposure. (D) ABR recordings obtained at day 0 prior to acoustic over-exposure reveal a hearing threshold of 34 dB SPL at 16 kHz. (E) ABR recordings obtained 4 days after acoustic over-exposure reveal a hearing threshold of 81 dB SPL at 16 kHz. (F) Summary graph shows significantly elevated hearing thresholds for frequencies exceeding 8 kHz, 4 days after acoustic over-exposure (grey diamonds) in comparison to hearing thresholds obtained at day 0, prior to acoustic over-exposure (black squares). *N* = 8, (C,F) ***P < 0.001, paired *t*-tests, ns: non-significant.

Exposure to loud sound leads to hearing loss in animals and humans (Daniel, 2007, Moser et al., 2013, Zhao et al., 2010) and to a plethora of neural changes in the DCN which correlates with the behavioural evidence of tinnitus (reviewed in Eggermont and Tass, 2015, Shore et al., 2016). We postulated that early functional deficits in synaptic transmission and plasticity at DCN multisensory synapses following acoustic over-exposure represent the earliest triggers for subsequent deficits leading to tinnitus. We also hypothesised that their identification could allow early interventions delaying or alleviating the onset of tinnitus following hearing loss. Effects of acoustic over-exposure (110 dB SPL, 14.8 kHz for 9 h) on auditory brainstem responses thresholds were assessed 4 days after insult. Whereas no change in hearing threshold was observed at day 0 and day 4 in control animals **(**Fig. 3A–C), acoustic over-exposure increased hearing thresholds by 30–60 dB SPL (Fig. 3D–F) for frequencies above the frequency of insult i.e. 16 kHz as previously reported (Pilati et al., 2012a).

**Fig. 4:**
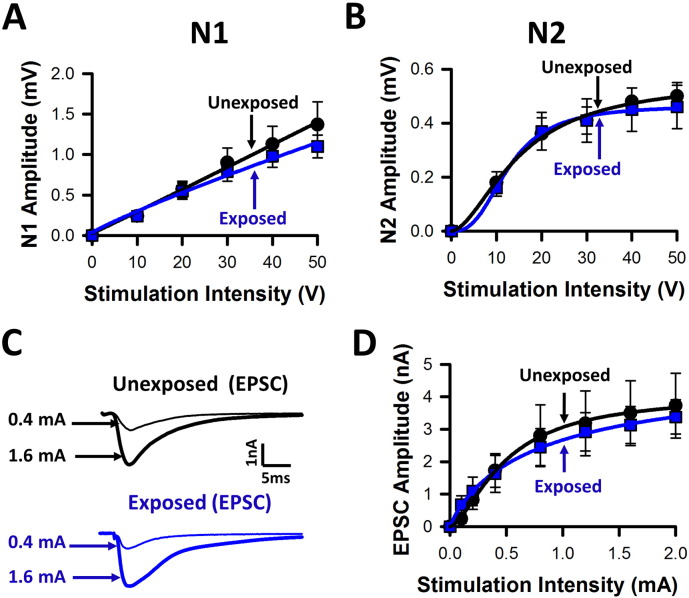
Absence of effects of acoustic over-exposure on basal synaptic transmission. Absence of effects of acoustic over-exposure on (A) the presynaptic field potential amplitude (N1) and (B) the post-synaptic field potential amplitude (N2). (A, B) Amplitudes were recorded in response to graded stimulation intensities and fitted with a Hill function in both the unexposed condition (black circles, n = 11, N = 11) and during hearing loss after acoustic over-exposure (blue squares, n = 9, N = 6). (C) Absence of effects of acoustic over-exposure on excitatory post-synaptic currents (EPSCs) recorded in fusiform cells. Average of 10 EPSCs evoked at basal stimulation rates in the unexposed condition (above traces) and during hearing loss (below traces). EPSCs were evoked at a stimulation intensity of 0.4 mA (thin traces) and 1.6 mA (thick traces) as indicated. (D) Input-output relationships of the EPSCs evoked at various current stimulation intensities were fitted with a Hill function in the unexposed (black circles, n = 9, N = 8) and exposed (blue squares, n = 9, N = 7) conditions.

**Fig. 5:**
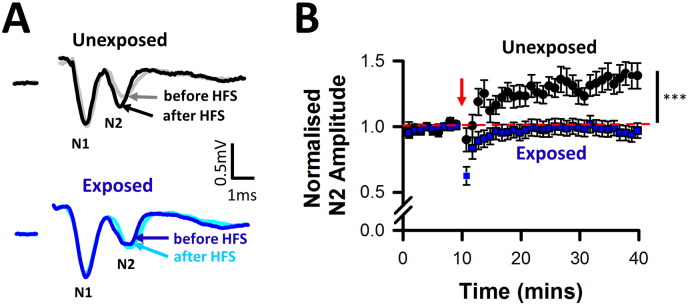
Metaplasticity following acoustic over-exposure. (A) Average of 10 field potentials recorded before high frequency stimulations (HFS, grey and blue) and 30 min after HFS (black and cyan) in the unexposed condition (above) and during hearing loss after acoustic over-exposure (below). Stimulation artefacts were deleted for clarity. (B) High frequency stimulations (red arrow) induced LTP of the PSFPs in unexposed conditions. Thirty minutes after HFS, PSFP (N2) amplitudes were potentiated by 39 ± 09% (black circles, n = 16, N = 15; Z = 3.46, Wilcoxon P < 0.001). High frequency stimulations failed to elicit LTP after acoustic over-exposure (normalised PSFP amplitude of 0.96 ± 0.06; n = 20, N = 13, Z = − 0.63, Wilcoxon P = 0.55, blue squares). ***P < 0.001, U = 47, Mann Whitney test comparing PSFPs after HFS in the unexposed and exposed condition. The red dashed line indicates the baseline amplitude.

We investigated basal synaptic transmission at DCN multisensory synapses in brainstem slices 4–5 days after acoustic over-exposure. Postsynaptic field potentials elicited at basal stimulation rates (0.3 Hz) displayed similar amplitudes to those recorded in unexposed conditions, for both the presynaptic volley (N1, Fig. 4A) and the PSFP (N2, Fig. 4B). Excitatory postsynaptic currents (EPSCs) evoked in identified fusiform cells by parallel fiber stimulations were also of similar amplitude between the two conditions (Fig. 4 C, D), confirming that basal synaptic transmission at DCN multisensory synapses was unaffected during hearing loss. By contrast to the absence of effect on basal synaptic transmission, acoustic over-exposure altered synaptic plasticity, as HFS leading to LTP in unexposed conditions failed to induce LTP during hearing loss (Fig. 5 A, B). Acoustic over-exposure therefore alters synaptic plasticity at DCN multisensory synapses, providing evidence of metaplasticity (Yger and Gilson, 2015).

**Fig. 6:**
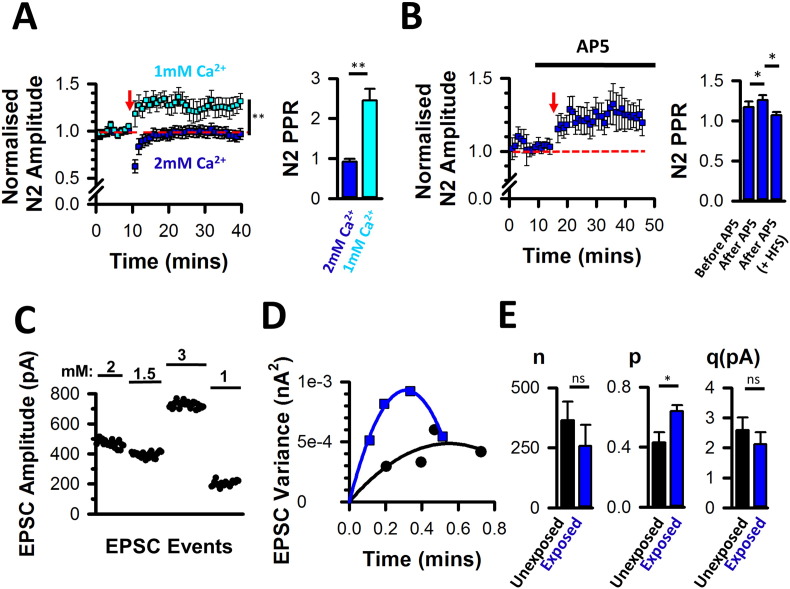
Metaplasticity is related to high release probability. (A left) Lowering Ca^2 +^ concentration to 1 mM allowed LTP to be induced following acoustic over-exposure (normalised PSFP amplitudes increasing from 1.01 ± 0.01 to 1.27 ± 0.09 after high frequency stimulations , n = 11, N = 5; Z = 2.93, P = 0.02, Wilcoxon). PSFP amplitudes were significantly higher than values recorded 30 min after high frequency stimulation in 2 mM Ca^2 +^ (n = 20, N = 13, U = 47, *P = 0.01 Mann Whitney test comparing PSFPs measured after HFS in the two conditions). (A right) Decreasing extracellular Ca^2 +^ from 2 mM to 1 mM increased paired pulse ratios from 0.97 ± 0.07 to 2.45 ± 0.29 (n = 4, N = 2, Wilcoxon, P < 0.01). (B left) Induction of LTP in the presence of 25 μM D-AP5 (normalised PSFPs increasing from 1.02 ± 0.01 to 1.23 ± 0.09 after high frequency stimulations (n = 14; N = 9; P = 0.02, RM ANOVA on Ranks, n = 14, N = 9). (B right) Increased paired pulse ratios in the presence of D-AP5 at baseline stimulations rates and decreased paired pulse ratios during LTP (from 1.26 ± 0.06 to 1.07 ± 0.04 after high frequency stimulations, n = 7; N = 5; P = 0.007, RM ANOVA on Ranks, SNK test). (C) Scatter plot of the EPSC amplitudes measured in various extracellular Ca^2 +^ concentrations (in mM) in the unexposed condition, as part of the variance-mean analysis. (D) Two examples of EPSC variance-mean plots fitted with a parabola function (unexposed: black circle; acoustic over-exposure: blue square). (E) Acoustic over-exposure increased the release probability (t(16) = − 2.6 P = 0.019, unpaired *t*-test) while leaving the quantal size (q; unexposed: 2.6 ± 0.4; exposed: 2.1 ± 0.4; t(16) = 0.8; *P* = 0.43, unpaired *t*-test) and the number of release sites (n; unexposed: 365 ± 78; exposed: 257 ± 89; t(16) = 0.9; P = 0.38, unpaired *t*-test) unaffected. Unexposed: n = 9, N = 9; Exposed: n = 9, N = 7.

Having previously demonstrated that a low release probability is a prerequisite for LTP induction (Fig. 2A), we hypothesised that the absence of LTP during hearing loss was due to an increased release probability at DCN multisensory synapses and that decreasing the release probability should restore the LTP induction in response to HFS. Decreasing extracellular Ca^2 +^ concentration from 2 mM to 1 mM indeed promoted paired pulse facilitation (Fig. 6A right) and restored LTP induction after acoustic over-exposure (Fig. 6A left). In summary, following acoustic over-exposure, brainstem slices failed to express LTP unless the release probability was lowered by decreasing extracellular calcium, a procedure similarly used by (Schulz, 1997) to demonstrate a presynaptic contribution to LTP at hippocampal synapses. LTP induction could also be restored by performing HFS in the presence of D-AP5 (Fig. 6B left). Similar to the unexposed conditions, the restoration of LTP by D-AP5 following acoustic over-exposure was accompanied by a decrease in paired pulse ratios, indicating an increased release probability (Fig. 6B right).

We have reported previously that acoustic over-exposure decreased the number of release sites at fusiform cell-auditory nerve synapses using the variance-mean method of quantal analysis (Tagoe et al., 2014). Similar quantal analysis of EPSCs evoked in identified fusiform cells by parallel fiber stimulations, confirmed an increased release probability at fusiform cell-multisensory synapses after acoustic over-exposure (Fig. 6C–E). In summary, four days after acoustic over-exposure, there is a frequency specific hearing loss and metaplasticity at DCN multisensory synapses resulting in an occlusion of LTP due to an increased release probability.

### 3.3 Administration of magnesium-l-threonate protects against metaplasticity and gap detection deficits after acoustic over-exposure

**Fig. 7:**
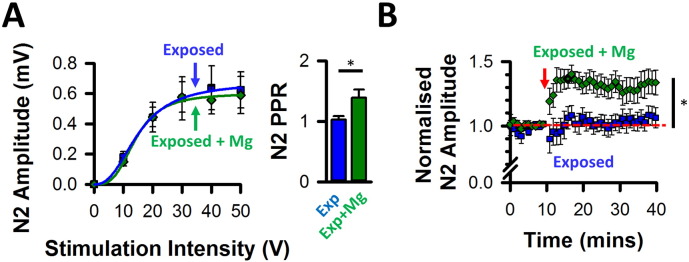
LTP induction after acoustic over-exposure following enriched magnesium diet. (A) Absence of effect on basal synaptic transmission. (A left) Similar PSFPs were obtained 4 weeks after acoustic over-exposure or when Mg^2 +^ was administered for 4 weeks after acoustic over-exposure (Exposed: blue squares, n = 7, N = 4; Exposed + Mg^2 +^: green diamonds, n = 9, N = 4). Relationships were fitted with a Hill function. (A right) In vivo administration of Mg^2 +^ after acoustic overexposure restored in vitro paired pulse facilitation under basal stimulating conditions (n = 6, N = 4; t (5) = 3.5, P = 0.02, paired *t*-test comparing the two N2 amplitudes). Higher paired pulse ratios were also obtained in the “Exposed + Mg^2 +”^ group in comparison to the “Exposed” group (Exposed: n = 8, N = 6; Exposed + Mg^2 +^: n = 6, N = 4; Mann Whitney, U = 8, *P < 0.05). (B) LTP was absent 4 weeks after acoustic over-exposure and was restored when Mg^2 +^ was administered immediately after acoustic over-exposure and for a period of 4 weeks (comparing normalised amplitudes before and after high frequency stimulations: Exposed (blue squares), n = 6, N = 4; Z = 0.9, NS, Wilcoxon Exposed + Mg^2^ (green diamonds), n = 12, N = 5; Z = 2.46, P = 0.008, Wilcoxon;). PSFP amplitudes measured 30 min after high frequency stimulations, were different in the two conditions (Mann Whitney, U = 10, *P = 0.024).

Previous studies showed that administration of Mg^2 +^-threonate reduced the release probability at hippocampal synapses, enhancing both short-term synaptic facilitation and LTP, in addition to improving the function of learning and memory (Slutsky et al., 2010). We similarly administered Mg^2 +^-threonate for one month after acoustic over-exposure and tested the effects on synaptic plasticity at DCN multisensory synapses. In vivo administration of Mg^2 +^-threonate failed to affect PSFPs evoked at basal stimulation rates (Fig. 7A left). Similarly, Slutsky et al. (2010) found no effect on basal synaptic transmission in hippocampal slices from Mg^2 +^-threonate-treated rats. By contrast, Mg^2 +^-threonate administration promoted paired pulse facilitation (Fig. 7A right) and restored the induction of LTP after acoustic over-exposure (Fig. 7B). This suggests that in vivo administration of Mg^2 +^-threonate decreased the release probability, thereby restoring LTP at DCN multisensory synapses.

**Fig. 8:**
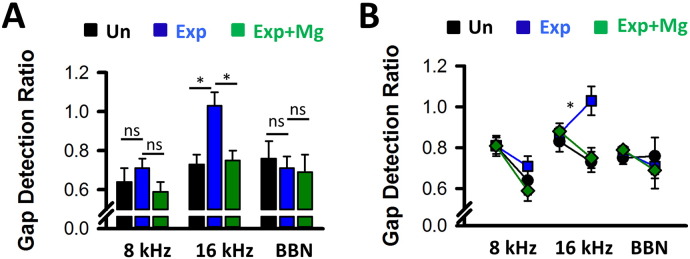
Effects of enriched magnesium diet on gap detection ratios following acoustic over-exposure. (A) Eighteen weeks after acoustic over-exposure, silent gaps were detected when embedded in broadband noise (BBN) or in 8 kHz background sound but were undetected when embedded in a 16 kHz background sound (N = 9). However, administration of Mg^2 +^ following acoustic over-exposure prevented gap discrimination deficits at 16 kHz (N = 9, *P < 0.05 One Way ANOVA). (B) Gap detection ratios for 8 kHz, 16 kHz and BBN calculated for week 0 (left symbols) and week 18 (right symbols) show that Mg^2 +^ administration prevented gap discrimination deficits otherwise present at 16 KHz at week 18 after AOE (*P < 0.05, linear mixed model, pairwise comparison; unexposed: N = 7; Exposed: N = 9; Exposed + Mg^2 +^: N = 9).

Numerous studies have shown that acoustic over-exposure affects the gap-prepulse inhibition of the acoustic startle reflex, a broadly applied paradigm to study changes in neural processing related to tinnitus (Engineer et al., 2011, Longenecker et al., 2014, Turner and Larsen, 2016). We assessed gap detection deficits 18 weeks after acoustic over-exposure, when auditory brainstem thresholds had recovered to values below 65 dB SPL at 8 kHz, 16 kHz and broadband noise frequency (Table 1), and observed a deficit in detecting gaps at 16 kHz (Table 1). Administration of Mg^2 +^-threonate abolished the deficits in gap detection at 16 kHz (Table 1, Fig. 8A). We next performed a linear mixed model allowing evaluation of a potential time- dependent effect between week 0 and week 18 (after acoustic over-exposure) **(**Fig. 8B) and confirmed that the effect on gap detection ratios at 16 kHz was due to acoustic over-exposure and not due to time. In summary, administration of Mg^2 +^-threonate reverses both the deficits in LTP observed early after acoustic over-exposure, and gap detection deficits at a later stage.

## 4 Discussion

Our present study suggests links between tinnitus and metaplasticity at DCN multisensory synapses. Acoustic over-exposure leads to an increased release probability and this saturates LTP at these synapses. Multisensory inputs into the DCN are carried by parallel fibers which form synapses onto a variety of cell types, including principal fusiform and inhibitory cartwheel cells (Oertel and Young, 2004). Metaplasticity observed here using field potential recordings could therefore result from plastic alterations in fusiform or cartwheel cells as both cell types undergo LTP (Fujino and Oertel, 2003). Our whole cell recordings of identified fusiform cells followed by a variance-mean method of quantal analysis on evoked EPSCs confirmed an increased release probability at parallel fiber-fusiform cell synapses after acoustic over-exposure, suggesting that plasticity is also likely to be altered in these cells. Interestingly, an increased release probability could be directly linked to the increased expression of VGluT2 glutamate transporters observed after acoustic trauma (Barker et al., 2012, Shore et al., 2008, Zhou et al., 2007), with VGluT2 being associated with higher release probability in comparison to VGluT1 that is also expressed at the synapse (Fremeau et al., 2004). A greater release probability has also been reported in the anteroventral cochlear nucleus of deaf mice (Oleskevich et al., 2000), possibly as a compensatory mechanism (Davis and Bezprozvanny, 2001) to the decreased synaptic activity at auditory synapses (Tagoe et al., 2014). Studies show that acoustic over-exposure triggers synaptic terminal swelling in the cochlea that could be partially blocked by perfusion of glutamate receptor antagonists (Pujol et al., 1985, Pujol and Puel, 1999). Acoustic overexposure also damages ribbon synapses to inner hair cells, causing delayed degeneration of auditory nerve fibers (Kujawa and Liberman, 2009). How metaplasticity at DCN multisensory synapses links to cochlear damage has yet to be determined but could be related to dysfunctional auditory synaptic integration within the DCN or via the auditory cortex sending descending inputs to the granule layer (Weedman and Ryugo, 1996).

NMDA receptors are known to be essential triggers for LTP at many excitatory synapses (Malenka and Bear, 2004) although they are not involved in LTP induction in DCN cartwheel cells or in a proportion of fusiform cells (Fujino and Oertel, 2003). In our control brainstem slices, blocking NMDA receptors did not measurably affect PSFPs recorded at low stimulation rates, most likely because the depolarization reached at this stimulation frequency is insufficient to relieve NMDA receptors from the Mg^2 +^ block (Ascher and Nowak, 1988). Blocking NMDA receptors inhibits the maintenance but not the induction of LTP at DCN multisensory synapses, suggesting a downstream event following high frequency stimulations, which is likely to relieve NMDA receptors from the Mg^2 +^ block, and facilitates the LTP induction. By contrast, prolonged exposure to NMDA or blocking glutamate uptake increases the release probability and abolishes the LTP induction. These findings suggest that NMDA receptors are likely to act as biosensors of extracellular glutamate, participating in the presynaptic modulation of LTP in the DCN.

Given the well-established role of NMDA receptors in long-term neuroplasticity (Bear and Malenka, 1994, Bliss and Collingridge, 1993, Tsien, 2000), their participation in metaplasticity within the DCN following acoustic over-exposure was anticipated. A similar saturation phenomenon of LTP has been described in mice in which NMDA receptor subunit combinations were altered leading to reductions in contextual learning (Kiyama et al., 1998) and in a murine model of Rett syndrome caused by mutations in the X-linked gene MECP2 (Weng et al., 2011). In both cases, application of an NMDA receptor blocker resulted in partial restoration of LTP (Kiyama et al., 1998, Weng et al., 2011). In the visual system, sensory deprivation triggers metaplasticity depending on NR2A/NR2B NMDA receptor subunit ratios (Philpot et al., 2003, Philpot et al., 2001) and reinstates presynaptic NMDA receptor-mediated plasticity (Larsen et al., 2014). Our study identifies a role of NMDA receptor activation in the presynaptic modulation of LTP and hence in metaplasticity in the DCN after acoustic over-exposure. However it also leaves some unanswered questions, in particular, whether glutamate acts as a retrograde messenger on pre-synaptic NMDA receptors (Tzingounis and Nicoll, 2004) and/or whether this pre-synaptic form of LTP involves other forms of retrograde signalling (Tzounopoulos et al., 2007) or changes in NMDA receptor subunit compositions (Cui et al., 2009) modulating glutamate release probability and affecting temporal processing (Sun et al., 2011).

Magnesium-l-threonate has previously been shown to efficiently increase brain Mg^2 +^, reducing release probability and abolishing the impairment of LTP at hippocampal synapses (Slutsky et al., 2010), and restoring short-term memory deficits associated with neuropathic pain (Wang et al., 2013). In the present study, Mg^2 +^-threonate was administered after acoustic over-exposure, reducing release probability and restoring LTP at DCN multisensory synapses. Although cellular mechanisms underlying the effects of Mg^2 +^ are still poorly understood, its potent blocking action of NMDA receptors (Traynelis et al., 2010) could be central to understanding its role in preventing the synaptic deficits induced by acoustic over-exposure in the DCN.

**Fig. 9:**
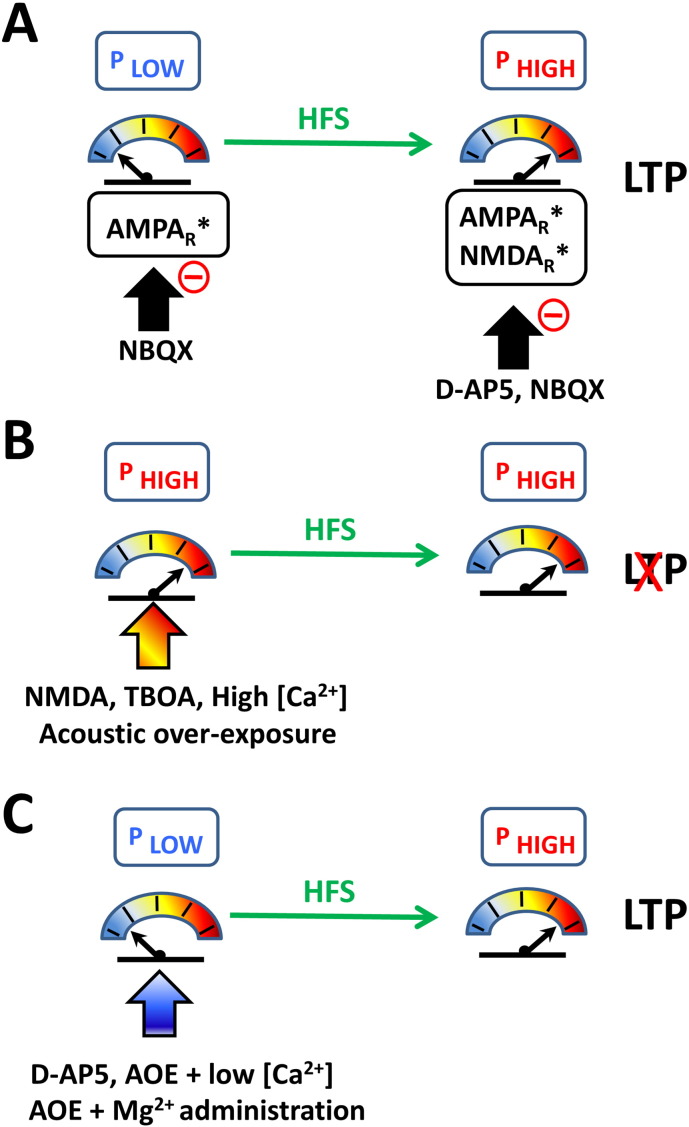
Schematic summarizing the modulation of LTP at DCN multisensory synapses in function of the release probability (P). (A) Basal stimulation of parallel fibers leads to activation of AMPA receptors (AMPA_R_*) with PSFPs blocked by NBQX. High frequency stimulations (HFS) triggers LTP via AMPA and NMDA receptor activation (AMPA_R_* and NMDA_R_*), with PSFPs inhibited by NBQX and D-AP5 respectively. LTP is dependent on an initial low release probability (P_LOW_). The release probability increases after high frequency stimulations (P_HIGH_). (B) NMDA receptor activation, glutamate uptake inhibition, high Ca^2 +^ concentration or acoustic over exposure increase the release probability and triggers metaplasticity. (C) NMDA receptor inhibition, lowering Ca^2 +^ concentration after acoustic over-exposure (AOE), or administration of Mg^2 +^ after acoustic over-exposure decrease the release probability and allows LTP induction after acoustic over-exposure.

We used the deficit in the gap-induced prepulse inhibition of the acoustic startle reflex as a behavioural sign of tinnitus and demonstrated gap detection deficits 18 weeks after acoustic over-exposure; and showed that administration of Mg^2 +^-threonate abolished these gap detection deficits. Despite possible interpretations linked to gap detection deficits in a murine model, our study supports previous conclusions reporting that Mg^2 +^ supplementation decreases the tinnitus perception in patients with moderate to severe tinnitus (Cevette et al., 2011). It is possible that the effectiveness of Mg^2 +^-threonate could decline if it were to be administered outside a “consolidation window” following acoustic trauma (Guitton and Dudai, 2007). Nonetheless, our study demonstrates a pathological metaplasticity in the auditory brainstem (summarised in Fig. 9) that could be abated with the administration of Mg^2 +^-threonate.

## Disclosures

No conflicts of interest, financial or otherwise, are declared by the author(s).

## Figures and Tables

**Table 1:** Effects of Mg^2 +^ on hearing thresholds (dB SPL) and gap detection ratios, measured 18 weeks after acoustic over-exposure. Hearing thresholds and gap detection ratios are assessed at 8 kHz, 16 kHz and broadband noise (BBN). Hearing thresholds are under 65 dB SPL for all experimental conditions (Unexposed: Un, Exposed: Exp and Exposed + Mg^2 +^). One way ANOVA or ANOVA on Ranks tests were used to assess overall conditions (last row). Student-Newman-Keuls post hoc or Dunn's post hoc tests were used to perform pairwise comparisons (when an overall difference was assessed). Gap detection ratios show higher values for 16 kHz in the exposed group (indicating deficit at that frequency). Magnesium administration lowered this ratio to values similar to those obtained in the unexposed condition.

	Hearing thresholds (dB SPL)			Gap detection ratio		
	8 kHz	16 kHz	BBN	8 kHz	16 kHz	BBN
Unexposed (N = 7)	38.7 ± 3.2	37.3 ± 5.7	34.3 ± 4.1	0.64 ± 0.07	0.73 ± 0.05	0.76 ± 0.09
Exposed (N = 9)	50.4 ± 4.2 vs Un: P = 0.032	43.2 ± 4	35.4 ± 2.6	0.71 0.05	1.05 ± 0.08 vs Un: P < 0.05	0.71 ± 0.06
Exposed + Mg^2 +^ (N = 9)	49.9 ± 3.8 vs Exp: P = 0.86 vs Un: P = 0.018	46.8 ± 1.5	41.7 ± 1.6	059 ± 005	0.71 ± 0.06 vs Exp: P < 0.05 vs Un: NS	0.69 ± 0.09
One way ANOVA tests	F(2) = 4.4 P = 0.02⁎	F(2) = 1.9 P = 0.18	F (2) = 2.1 P = 0.11	(on Ranks) H(2) = 1.1; P = 0.59	(on Ranks) H(2) = 9.2; P = 0.01⁎	(on Ranks) H(2) = 0.58; P = 0.7

⁎ P < 0.05.
